# Nutritional Biomarkers, Bone Turnover, and Oxidative DNA Damage in Postmenopausal Women with Periodontitis: A Cross-Sectional Study

**DOI:** 10.3390/nu18050845

**Published:** 2026-03-05

**Authors:** Irina-Georgeta Sufaru, Stefan-Lucian Burlea, Maria-Alexandra Martu, Sorina Mihaela Solomon, Maria-Georgeta Laza, Liliana Pasarin, Alexandra Cornelia Teodorescu, Ioana Martu

**Affiliations:** Grigore T. Popa University of Medicine and Pharmacy, 700115 Iasi, Romania; ursarescu.irina@umfiasi.ro (I.-G.S.); maria-alexandra.martu@umfiasi.ro (M.-A.M.); sorina.solomon@umfiasi.ro (S.M.S.); laza.gina11@gmail.com (M.-G.L.); liliana.pasarin@umfiasi.ro (L.P.); cornelia.teodorescu@umfiasi.ro (A.C.T.); ioana.martu@umfiasi.ro (I.M.)

**Keywords:** bone mineral density, osteoporosis, oxidative DNA damage, periodontitis, vitamin D

## Abstract

**Background/Objectives:** Periodontitis and low BMD often occur after menopause, but the role of nutritional status in the oral–skeletal link is unclear. This study examined whether nutritional biomarkers relate to periodontitis severity and modify the relationship between low BMD and periodontal destruction in postmenopausal women. **Methods:** This cross-sectional study included 120 postmenopausal women who underwent comprehensive periodontal measurements at six sites per tooth and were classified according to the 2017 World Workshop staging and grading framework. Areal BMD was measured using dual-energy X-ray absorptiometry (DXA). Objective biomarkers included serum 25(OH)D, plasma vitamin C, RBC omega-3 index, and serum ferritin. Mechanistic measures were serum CTX, P1NP, and urinary 8-OHdG/creatinine. The main periodontal outcome was the mean CAL. **Results:** Low BMD was associated with greater periodontal destruction (mean CAL 2.06 vs. 1.45 mm; adjusted β = 0.664 mm, 95% CI 0.465–0.863; *p* < 0.001). Higher 25(OH)D and omega-3 index were independently associated with lower mean CAL (β = −0.024 mm per 1 ng/mL and β = −0.107 mm per 1%, respectively), with false discovery rate control applied across nutritional biomarkers. Across the cohort, serum 25(OH)D showed a weak inverse correlation with CTX (*r* = −0.14; *p* = 0.141), and exploratory mediation analyses suggested only small indirect effects via CTX and 8-OHdG. **Conclusions:** In women after menopause, lower BMD is associated with greater periodontal tissue loss. Objective nutritional biomarkers, especially 25(OH)D and omega-3 levels, correlate with biologically plausible pathways involved in periodontal destruction and remodeling. This supports the idea that nutrition could be a key factor linking oral health and skeletal health.

## 1. Introduction

Periodontitis and osteoporosis are among the most common chronic diseases linked to aging [1], posing a significant global health challenge. Current Global Burden of Disease reports reveal that oral health issues are widespread worldwide, with periodontitis notably contributing to disability and tooth loss in adults [2]. Similarly, postmenopausal bone loss and osteoporosis are prevalent, have critical clinical impacts, and are heavily influenced by hormonal and lifestyle changes following menopause [3]. The simultaneous presence of periodontal tissue damage and low bone mineral density (BMD) in postmenopausal women has attracted increasing interest because both share key features—bone resorption, structural weakness, and age-related progression—though their exact relationship and underlying mechanisms remain poorly understood [4].

Recent systematic reviews and meta-analyses support a link between low BMD/osteoporosis and poorer periodontal health, though results are often affected by differences in periodontal case definitions, incomplete skeletal phenotyping, and inconsistent control of confounding factors [4,5,6,7]. A key methodological challenge in the broader research is the use of simplified periodontal indicators or partial-mouth assessments, which can underestimate disease presence and make cross-study comparisons difficult. The 2017 World Workshop classification provides a clinically relevant staging and grading system that integrates severity, complexity, and risk/progression factors, offering a more standardized approach for mechanistic studies and cohort comparisons [8].

Nutrition is a promising candidate for explaining the variability in the relationship between osteoporosis and periodontitis [9] because it is biologically plausible, modifiable, and measurable using objective biomarkers. In particular, vitamin D status lies at the crossroads of skeletal mineral metabolism and periodontal tissue biology, and updated meta-analyses continue to show that individuals with periodontitis tend to have lower circulating vitamin D levels compared to controls [10]. Broader research on vitamin intake and periodontal health further suggests that adequate micronutrient intake correlates with better periodontal outcomes, though observational studies remain prone to confounding and measurement error when relying solely on questionnaires for intake estimates [11,12,13,14].

Nutrients with antioxidant properties are also relevant because periodontal tissue breakdown involves connective tissue degradation and alveolar bone loss in an oxidative environment [15]. Systematic review evidence indicates that vitamin C intake or supplementation is associated with periodontal benefits in certain clinical contexts, despite notable heterogeneity [16,17]. Recent reviews further support a broad role for nutrition in periodontal health, emphasizing that micronutrient and omega-3 fatty acid deficiencies may contribute to impaired host response, oxidative stress, and reduced periodontal tissue repair, while also highlighting the need for more well-controlled clinical and biomarker-based studies [18]. Additionally, omega-3 fatty acids have been studied as adjuncts to non-surgical periodontal therapy, with systematic reviews and meta-analyses finding modest additional improvements in probing depth and clinical attachment compared to therapy alone [19,20,21]. Overall, this evidence supports the idea that nutritional status can directly influence periodontal health and may also alter the manifestation of skeletal fragility in periodontal tissues.

A persistent issue is that many oral–skeletal studies rely primarily on self-reported diet questionnaires, while mechanistic panels often focus on systemic inflammatory markers that may overlap with those in other immunology research. Using objective, clinic-friendly biomarkers offers an alternative means of achieving mechanistic specificity. For instance, urinary oxidative DNA damage markers have been tested in periodontitis biomarker studies, demonstrating their feasibility and potential significance [22]. The marker 8-hydroxy-2′-deoxyguanosine (8-OHdG) is discussed as a reliable indicator of oxidative damage in periodontal research [23,24]. Similarly, bone turnover markers like CTX and P1NP can reveal systemic remodeling processes relevant to both skeletal and alveolar bones [25,26,27].

Although there is increasing evidence of a link between osteoporosis, low BMD, and periodontitis in postmenopausal women, several gaps remain in the research. Many studies rely on simplified definitions of periodontal disease or assess only parts of the mouth, and nutritional exposure is often measured using self-reports rather than objective biomarkers. Moreover, skeletal phenotyping, periodontal staging, and detailed biomarker panels are rarely combined within a single cohort, which restricts the ability to explore the oral–skeletal relationship within a consistent biological framework. To fill these gaps, this study combined DXA-based BMD measurement with comprehensive periodontal phenotyping based on the 2017 World Workshop classification, along with an objective panel of biomarkers. Conducted in a carefully selected group of postmenopausal women, this integrative approach aimed to explore whether nutritional biomarkers are associated with periodontitis severity and whether they influence the variability in the relationship between low BMD and periodontal tissue destruction.

In this study, we adopted a focused conceptual framework that emphasizes variables directly measured in the cohort. We hypothesized that lower BMD would correlate with increased periodontal tissue destruction. Additionally, we examined objective nutritional biomarkers—such as 25(OH)D, plasma vitamin C, RBC omega-3 index, and ferritin—to determine whether they relate to periodontal severity and might help explain some of the variability in the oral–skeletal relationship. To delve deeper into the underlying mechanisms, we included bone turnover markers (CTX, P1NP) and urinary 8-OHdG/creatinine as indicators of remodeling activity and oxidative DNA damage, respectively, to determine whether the nutrition–periodontitis links are biologically consistent within this postmenopausal cohort.

## 2. Materials and Methods

### 2.1. Study Design and Reporting

This cross-sectional observational study examined whether objectively measured nutritional status influences the link between low bone mineral density (BMD) and the severity of periodontitis in postmenopausal women. The study employed periodontal phenotyping according to the 2017 World Workshop classification and evaluated biochemical markers related to nutritional pathways, including bone remodeling and redox balance. It adhered to the guidelines outlined by the Strengthening the Reporting of Observational Studies in Epidemiology (STROBE) statement.

### 2.2. Study Population, Recruitment, and Eligibility

Participants were consecutively recruited from the Clinic of Periodontology at Grigore T. Popa University of Medicine and Pharmacy in Iasi, Romania, from March 2024 to December 2025. Women eligible for the study were postmenopausal (defined as at least 12 months of amenorrhea not caused by other factors or having undergone bilateral oophorectomy), aged 50 to 75 years, and with at least 10 natural teeth (excluding third molars) to enable comprehensive full-mouth periodontal assessment.

The following exclusion criteria were applied: current or former smokers (including the history of smoking); pregnant or lactating women; those with metabolic bone diseases other than postmenopausal osteopenia or osteoporosis, such as hyperparathyroidism, osteomalacia, or Paget’s disease; individuals with stage 3 or higher chronic kidney disease; those with active malignancies; diabetics requiring insulin or with glycated hemoglobin (HbA1C) levels of 7.0% or higher; individuals with chronic inflammatory or autoimmune diseases; people with chronic liver disease; anyone with an acute infection or febrile illness at the time of sampling; those who received antibiotics in the past three months; individuals who underwent periodontal therapy in the last six months; patients with urgent current oral infections; those undergoing orthodontic treatment; and individuals who have used osteoporosis therapies (like bisphosphonates, denosumab, SERMs, calcitonin, teriparatide, abaloparatide, or romosozumab), menopausal hormone therapy, systemic glucocorticoids, proton-pump inhibitors, antidepressants (including SSRIs and SNRIs), or nutritional supplements (such as vitamin D, calcium, multivitamins or B-complex, omega-3 fatty acids, magnesium, zinc, iron, folate, or B12) within the last six months.

The study protocol received approval from the Institutional Review Ethics Committee of Grigore T. Popa University of Medicine and Pharmacy in Iasi, Romania (approval no. 406, dated 6 March 2024). All participants provided written informed consent prior to enrollment. Data were anonymized, securely stored on password-protected systems, and managed in accordance with relevant data protection laws.

### 2.3. Clinical Interview and Anthropometrics

A standardized interview and a medical record review were conducted to collect data on age, education level, residence (urban or rural), alcohol intake, physical activity, age at menopause, years since menopause, oral hygiene habits, dental visits, and medical history to verify eligibility. Height was measured with a wall-mounted stadiometer (Seca 217, seca GmbH & Co. KG, Hamburg, Germany), and weight was recorded with a calibrated digital scale (Seca 813, seca GmbH & Co. KG). Body mass index (BMI) was calculated in kg/m^2^. Blood pressure was measured twice after a 5 min rest with an automated monitor (OMRON M6 Comfort, Omron Healthcare, Kyoto, Japan), and the average of the two readings was recorded. Physical activity levels were assessed using the International Physical Activity Questionnaire—Short Form (IPAQ-SF), expressed in MET-min/week.

### 2.4. Periodontal Examination

Full-mouth periodontal examinations were conducted by calibrated periodontal specialists. Calibration sessions were completed prior to the commencement of the study with ten non-participant patients, involving duplicate measurements taken one week apart; a minimum intraclass correlation coefficient (ICC) of 0.80 for probing pocket depth (PPD) and clinical attachment level (CAL) was mandated before participants were enrolled. All teeth, excluding third molars, were examined at six sites per tooth—mesiobuccal, mid-buccal, distobuccal, mesiolingual, mid-lingual, and distolingual—using a UNC-15 periodontal probe (UNC-15, Hu-Friedy, Chicago, IL, USA). Parameters recorded included PPD (millimeters), gingival recession (millimeters), CAL (millimeters), bleeding on probing (BOP; yes/no within 15 s) [28], and plaque presence (O’Leary Plaque Control Record; yes/no per surface) [29]. Furcation involvement (Hamp classification) [30] and tooth mobility (Miller index) [31] were documented where present. The total number of teeth present was also recorded.

Periodontitis diagnosis, staging, and grading followed the 2017 World Workshop classification [8]. Staging considered interdental clinical attachment loss (CAL), radiographic bone loss (RBL), tooth loss due to periodontitis, and complexity factors; grading relied on indirect indicators such as bone loss relative to age, risk modifiers, and, when available, documented disease progression. Since smokers and patients with uncontrolled diabetes were excluded, grading was mainly based on bone loss relative to age and observed progression.

### 2.5. Dental Imaging and Radiographic Bone Loss Assessment

Digital panoramic radiographs were obtained with a Planmeca ProMax unit (Planmeca Oy, Helsinki, Finland). If the panoramic image quality was insufficient for site-specific evaluation, standard periapical radiographs were taken using an intraoral X-ray unit (Planmeca ProX intra, Planmeca Oy) with the paralleling technique. Radiographic bone loss was measured as the distance from the cemento–enamel junction (CEJ) to the alveolar crest at interproximal sites and/or as the percentage of root length involved, using Planmeca Romexis software (Romexis v.6.4.8, Planmeca Oy, Helsinki, Finland). Two independent, blinded examiners evaluated RBL; any disagreements were resolved through consensus. Inter-rater reliability was assessed using ICC.

### 2.6. Bone Mineral Density Assessment

Areal BMD (g/cm^2^) was assessed via dual-energy X-ray absorptiometry (DXA) at the lumbar spine (L1–L4) and proximal femur (including the total hip and femoral neck) with a GE Lunar iDXA system (GE Healthcare, Madison, WI, USA). Daily quality control involved using the manufacturer’s phantom. Scans were collected and analyzed following the guidelines of the International Society for Clinical Densitometry (ISCD) [32].

BMD status was categorized based on T-scores: normal (≥−1.0), low BMD (<−1.0), and osteoporosis (≤−2.5). For primary analysis, low BMD was defined as a T-score below −1.0 at either the lumbar spine or total hip/femoral neck (whichever was lower), and served as the main skeletal exposure. Additionally, continuous BMD values were utilized in secondary analyses.

### 2.7. Objective Nutritional Status Assessment

Participants attended a morning visit following an overnight fast of at least 8 h. Venous blood was drawn between 07:00 and 10:00, centrifuged within two hours, aliquoted, and stored at −80 °C until analysis. The sampling season was recorded in advance to account for seasonal differences in vitamin D levels.

Serum 25-hydroxyvitamin D [25(OH)D] levels were determined using electrochemiluminescence immunoassay (Elecsys Vitamin D total II, Roche Diagnostics, Mannheim, Germany) on a cobas e 411 analyzer (Roche Diagnostics). Plasma vitamin C (ascorbate) was quantified via high-performance liquid chromatography with UV detection after stabilization with metaphosphoric acid, employing an HPLC system (Agilent 1260 Infinity II, Agilent Technologies, Santa Clara, CA, USA).

The RBC omega-3 index (EPA + DHA as % of total identified fatty acids) was assessed by gas chromatography following fatty-acid methyl ester derivatization (Agilent 7890B GC system, Agilent Technologies). Serum ferritin was measured through electrochemiluminescence immunoassay (Elecsys Ferritin, Roche Diagnostics) on the cobas e 411 platform; ferritin was analyzed both as a continuous variable and, for secondary analysis, by tertiles.

Vitamin D levels were categorized as deficiency (<20 ng/mL), insufficiency (20–29 ng/mL), and sufficiency (≥30 ng/mL). However, the primary analyses treated 25(OH)D as a continuous variable to maintain statistical power in the clinic-based cohort.

### 2.8. Bone Remodeling Biomarkers

Fasting bone turnover markers were measured to evaluate remodeling activity, which might connect nutritional status to BMD and periodontal damage. Serum C-terminal telopeptide of type I collagen (CTX; Elecsys β-CrossLaps, Roche Diagnostics) and serum procollagen type I N-terminal propeptide (P1NP; Elecsys total P1NP, Roche Diagnostics) were tested using the cobas e platform following standardized preanalytical procedures during fasting morning samples.

### 2.9. Oxidative DNA Damage Biomarker

Systemic oxidative stress was assessed by measuring urinary 8-hydroxy-2′-deoxyguanosine (8-OHdG). Spot urine samples were collected during the same morning visit, aliquoted, and stored at −80 °C. The 8-OHdG levels were quantified using a competitive ELISA kit (Cayman Chemical, Ann Arbor, MI, USA), with readings obtained from a microplate reader (BioTek Synergy H1, Agilent Technologies, Santa Clara, CA, USA). Urinary creatinine was measured enzymatically (Roche Diagnostics; cobas c platform), and 8-OHdG concentrations were expressed as ng/mg creatinine.

### 2.10. Contextual Dietary Assessment

Participants completed a short food-frequency screener to provide descriptive context for their biomarker-defined nutritional status, focusing on the main sources of vitamin D, vitamin C, and long-chain omega-3 fatty acids. This screener was used solely for descriptive purposes and did not determine exposures, as the main nutrition-related variables were based on objective biomarkers.

### 2.11. Outcomes

The primary periodontal outcome was the mean clinical attachment level (CAL, mm). Secondary outcomes included severe periodontitis (comparing Stage III/IV to Stage I/II), the percentage of sites with CAL ≥ 5 mm, and the percentage of sites with probing pocket depth (PPD) ≥ 6 mm. The primary skeletal exposure was low BMD (T-score < −1.0), with BMD also assessed as a continuous variable. Nutrition-related variables measured were serum 25-hydroxyvitamin D [25(OH)D], plasma vitamin C, the RBC omega-3 index, and serum ferritin. Mechanistic variables included serum CTX, serum P1NP, and urinary 8-hydroxy-2′-deoxyguanosine (8-OHdG), normalized to creatinine.

### 2.12. Statistical Analysis

Analyses were conducted using Python (v.3.14, Python Software Foundation, Wilmington, DE, USA). Continuous variables are presented as mean ± standard deviation (SD) or median (interquartile range, IQR), as appropriate, and categorical variables are presented as n (%). Group comparisons between women with low versus normal BMD were performed using Welch’s *t*-test for approximately normally distributed variables, the Mann–Whitney U test for skewed variables, and the χ^2^ test for categorical variables.

Multivariable analyses were prespecified and organized by analytic objective. The primary inferential models evaluated associations between low BMD (main skeletal exposure), nutrition-related biomarkers, and periodontal outcomes, with mean clinical attachment level (CAL, mm) as the primary periodontal outcome. Secondary periodontal outcomes included severe periodontitis (Stage III/IV vs. Stage I/II), the percentage of sites with CAL ≥ 5 mm, and the percentage of sites with probing pocket depth (PPD) ≥ 6 mm. Linear regression models were fitted with heteroskedasticity-consistent HC3 robust standard errors.

Covariates were selected a priori on clinical and methodological grounds, based on their plausibility as confounders of the BMD–periodontitis and nutrition–periodontitis associations, and were retained consistently across the main models. The prespecified covariate set included age, body mass index (BMI), HbA1c, plaque burden (% plaque-positive sites), preventive dental attendance (≥1 visit/year), educational level, and place of residence. Covariate selection was not based on stepwise or significance-driven procedures. In models involving serum 25-hydroxyvitamin D [25(OH)D], sampling season was additionally included.

To preserve interpretability and minimize multicollinearity, nutrition-related biomarkers (serum 25(OH)D, plasma vitamin C, RBC omega-3 index, and serum ferritin) were analyzed as separate exposures in dedicated multivariable models (i.e., one biomarker per model), each adjusted for the same prespecified covariates. Because serum ferritin showed a right-skewed distribution, ferritin values were log-transformed before regression analyses to improve distributional symmetry and reduce the influence of high-value observations. False discovery rate (FDR) correction using the Benjamini–Hochberg procedure was applied only to this prespecified nutrition biomarker family, and adjusted results are reported as q-values. For primary analyses, statistical significance was set at *p* < 0.05. For the prespecified nutrition biomarker family, FDR-adjusted results were considered statistically significant at *q* < 0.05.

A separate interaction model tested whether serum 25(OH)D modified the association between BMD status and mean CAL by including a low BMD × centered 25(OH)D interaction term; the interaction coefficient and adjusted predicted values were reported. Severe periodontitis (Stage III/IV vs. Stage I/II) was analyzed as a secondary endpoint using multivariable logistic regression (binomial generalized linear model) with HC3 robust standard errors. Given the limited number of severe periodontitis events, the logistic regression results were interpreted as supportive/exploratory and with caution regarding precision.

Mechanistic biomarkers (CTX, P1NP, and urinary 8-hydroxy-2′-deoxyguanosine [8-OHdG]/creatinine) were treated as exploratory mechanistic readouts and analyzed in separate models using nominal *p*-values. CTX and P1NP were interpreted as downstream remodeling markers rather than alternative skeletal exposures and were therefore not entered simultaneously with BMD in the primary etiologic models. Exploratory mediation analyses evaluated CTX and urinary 8-OHdG/creatinine as potential statistical mediators of the association between 25(OH)D and mean CAL using a product-of-coefficients approach with nonparametric bootstrap confidence intervals (500 resamples). Because the study was cross-sectional, mediation analyses were considered hypothesis-generating and not evidence of temporally ordered causal pathways.

Secondary and exploratory analyses were interpreted primarily in terms of effect sizes and 95% confidence intervals. Model diagnostics were assessed for all main regression models. For linear models, residual plots (fitted vs. residuals and Q–Q plots) were examined, and HC3 robust standard errors were used as prespecified. For logistic models, model stability and influential observations were evaluated using standard diagnostic procedures. Multicollinearity was assessed using variance inflation factors (VIFs), and no evidence of problematic collinearity was identified (all VIFs within acceptable ranges).

### 2.13. Sample Size and Power Analysis

Sample size planning was constrained by the clinic-based recruitment setting and the strict eligibility criteria (including exclusion of current/former smokers, women receiving osteoporosis therapy or menopausal hormone therapy, and those using recent nutritional supplementation), which limited the feasible pool of eligible participants. Accordingly, the study was designed and powered primarily for the main-effect analysis of the primary continuous periodontal outcome, mean clinical attachment level (CAL), which is statistically more efficient than binary outcomes in cross-sectional designs.

Assuming a two-sided α of 0.05 and 80% power, a total sample size of N = 120 (with approximately balanced low-BMD and normal-BMD groups) provides 80% power to detect a Cohen’s d of approximately 0.52 for mean CAL (or other continuous periodontal measures) between BMD groups. This corresponds to a moderate standardized effect size (approximately 0.52 standard deviations).

The sample size calculation was not based on secondary outcomes or mechanistic analyses. In particular, the study was not specifically powered for event-limited logistic regression models of severe periodontitis (Stage III/IV), or for detecting small interaction (e.g., BMD × 25(OH)D) or small mediation effects (e.g., via CTX or urinary 8-OHdG/creatinine). Therefore, secondary, interaction, and mediation analyses were treated as supportive/exploratory and interpreted with appropriate caution, with emphasis on effect sizes and confidence intervals rather than statistical significance alone.

### 2.14. Data Quality and Missing Data

To ensure measurement reliability, all examiners participated in standardized training before the study began and conducted periodic calibration checks during recruitment. Calibration involved duplicate measurements of key periodontal parameters (PPD and CAL) on a subset of participants, with ongoing monitoring of agreement and retraining if drift occurred, supported by protocol reviews. Data collection adhered to predefined standard operating procedures, and clinical record forms were reviewed at the end of each visit to identify inconsistencies or missing fields before participants left.

Data were independently entered by two trained operators into a structured electronic database with predefined validity rules, and discrepancies were resolved by verifying source documents. Additional logic checks, including permissible ranges for probing measurements, internal consistency among CAL, recession, and PPD, and plausibility checks for DXA outputs and lab results, were applied. Laboratory analyses included batch-level quality control, with instrument calibration and control results reviewed before data analysis. The final dataset had no missing data.

All participants provided complete information for primary exposure variables (BMD status and continuous DXA measures), periodontal outcomes, biomarkers, mechanistic biomarkers, and covariates. Thus, analyses were conducted on the full sample (N = 120) using complete-case data without any imputation.

## 3. Results

### 3.1. Study Sample and Periodontal Classification

All 120 participants had complete clinical, DXA, and laboratory data (no dropouts). In the overall cohort (N = 120), Stage I periodontitis was most frequent (64.2%), followed by Stage III (22.5%) and Stage II (13.3%). The low-BMD group showed a higher proportion of Stage III disease (34.4%) than the normal-BMD group (8.9%), whereas Stage I disease was more common in the normal-BMD group (80.4% vs. 50.0%), indicating a shift toward more advanced periodontal destruction in women with low BMD. The distribution of periodontitis stages according to the 2017 classification is presented in Table 1.

### 3.2. Baseline Characteristics by BMD Status

Baseline demographic and clinical characteristics, stratified by BMD status, are summarized in Table 2. Mean age was 62.55 years in the normal-BMD group and 61.97 years in the low-BMD group; mean BMI was 25.76 vs. 27.69 kg/m^2^, respectively (Table 2).

Women with low BMD had a higher mean BMI than women with normal BMD (27.69 ± 3.48 vs. 25.76 ± 3.56 kg/m^2^, *p* = 0.004). Vitamin D status and RBC omega-3 index were lower in the low-BMD group, while CTX and urinary 8-OHdG/creatinine were higher, supporting a less favorable nutritional and mechanistic biomarker profile. Plaque and BOP percentages were similar between groups, suggesting that differences in periodontal destruction were not attributable to major differences in current plaque or bleeding burden (Table 2).

Within the low-BMD group (n = 64), 56 women (87.5%) had osteopenia and 8 women (12.5%) had osteoporosis (T-score ≤ −2.5), indicating that the low-BMD category was predominantly composed of osteopenic participants.

### 3.3. Periodontal Phenotype and Biomarker Profiles by BMD Status

Table 3 displays key periodontal measures, DXA metrics, and biomarkers categorized by BMD status. The average CAL was significantly higher in the low-BMD group (2.06 mm) compared to the normal-BMD group (1.45 mm; *p* < 0.001). Biomarkers related to nutrition also varied between groups, with the low-BMD group showing lower levels of 25(OH)D (18.14 vs. 22.66 ng/mL; *p* < 0.001), omega-3 index (4.45 vs. 5.32%; *p* < 0.001), and plasma vitamin C (43.28 vs. 49.85 µmol/L; *p* = 0.003; Table 3).

### 3.4. Multivariable Associations with Periodontal Destruction

In adjusted linear regression models with HC3-robust standard errors, low BMD was associated with higher average CAL (Table 4). The adjusted mean difference between low and normal BMD was β = 0.664 mm (95% CI 0.465 to 0.863; *p* < 0.001). Higher levels of 25(OH)D independently linked to reduced mean CAL (β = −0.024 mm per 1 ng/mL; 95% CI −0.041 to −0.008; *p* = 0.005; FDR *q* = 0.009).

Plasma vitamin C (β = −0.016 mm per 1 µmol/L; 95% CI −0.025 to −0.008; *p* < 0.001) and RBC omega-3 index (β = −0.107 mm per 1%; 95% CI −0.185 to −0.028; *p* = 0.008; FDR *q* = 0.011) were also inversely associated with mean CAL, whereas log-transformed ferritin was not (β = 0.016, 95% CI −0.162 to 0.194; *p* = 0.860; FDR *q* = 0.860; Table 4).

Model diagnostics did not indicate major violations of assumptions for the final regression models, and multicollinearity was not problematic based on variance inflation factor assessment. In practical terms, the adjusted low-BMD coefficient corresponds to an approximately 0.66 mm higher full-mouth mean CAL compared with normal BMD after covariate adjustment. Based on the fitted models, a 15 ng/mL higher serum 25(OH)D concentration corresponds to an estimated 0.36 mm lower mean CAL, whereas a 2% higher RBC omega-3 index corresponds to an estimated 0.21 mm lower mean CAL (Table 4).

**Figure 1:**
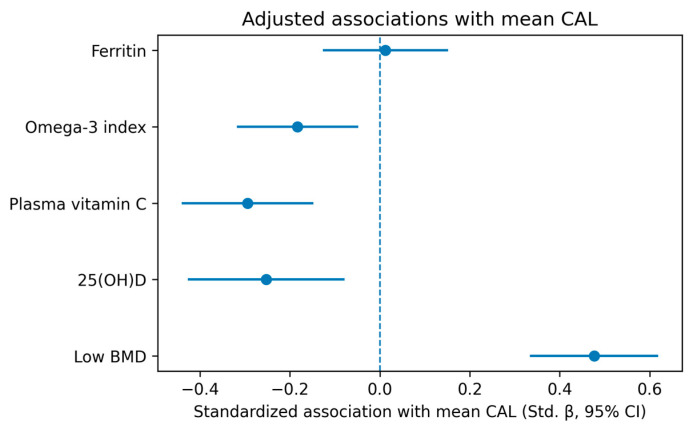
Adjusted effect estimates for the primary multivariable model (mean CAL).

Figure 1 summarizes the adjusted effect estimates and confidence intervals from the primary mean CAL model and visually confirms the direction and precision of the key associations reported in Table 4, with low BMD, lower 25(OH)D, lower vitamin C, and lower omega-3 index associated with greater periodontal tissue loss (Figure 1 and Table 4).

### 3.5. Effect Modification by 25(OH)D

**Figure 2:**
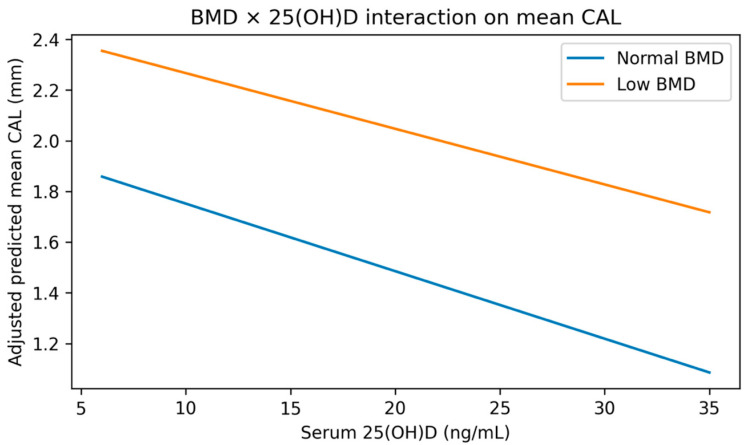
Adjusted predicted mean CAL across serum 25(OH)D values, stratified by BMD group. The near-parallel fitted lines in Figure 2 are consistent with the non-significant BMD × 25(OH)D interaction, indicating similar slopes across BMD strata.

To assess effect modification, a BMD × 25(OH)D interaction term was tested for mean CAL. The interaction coefficient was small and not statistically significant (β = 0.0047 mm per 1 ng/mL, 95% CI −0.0240 to 0.0334; *p* = 0.750), and adjusted predictions did not indicate meaningful differences in slope across BMD groups (Figure 2). Because the study was powered primarily for main-effect associations in continuous outcomes, this result argues against a large interaction effect but does not exclude more modest effect modification.

### 3.6. Secondary Endpoint: Severe Periodontitis (Stage III/IV)

In the multivariable logistic regression model for severe periodontitis (Stage III/IV vs. Stage I/II), low BMD was strongly associated with higher odds of severe disease (OR = 7.70, 95% CI 2.29–25.90; *p* < 0.001) (Table 5).

Increasing age was also independently associated with severe periodontitis (OR = 1.12 per year, 95% CI 1.01–1.24; *p* = 0.032), and higher plaque burden showed a robust association (OR = 2.00 per 10% increase in plaque-positive sites, 95% CI 1.32–3.04; *p* = 0.001).

In contrast, BMI was not associated with severe periodontitis (OR = 1.05 per kg/m^2^, 95% CI 0.89–1.24; *p* = 0.555). Preventive dental attendance (≥1 visit/year) showed a borderline inverse association (OR = 0.29, 95% CI 0.08–1.07; *p* = 0.063).

Higher vitamin D levels independently protected against severe periodontitis, showing significantly lower odds per 10 ng/mL increase in 25(OH)D (OR = 0.16, 95% CI 0.05–0.54; *p* = 0.003).

Although the association between low BMD and severe periodontitis was strong, the odds-ratio estimate should be interpreted cautiously because the number of Stage III/IV events was limited, which may reduce the estimate’s precision and model stability. Accordingly, this logistic analysis should be considered supportive of the primary continuous-outcome findings (mean CAL).

### 3.7. Exploratory Mechanistic and Mediation Analyses

**Figure 3:**
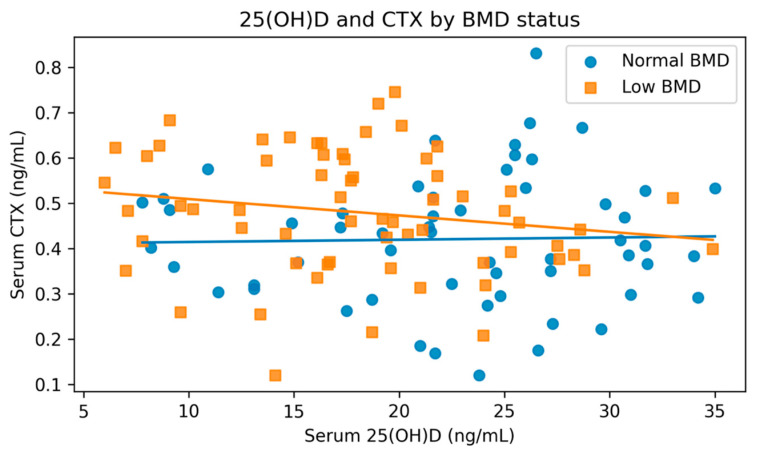
Scatter plot of serum 25-hydroxyvitamin D [25(OH)D] and serum CTX stratified by bone mineral density (BMD) status (normal vs. low BMD). Solid lines represent subgroup-specific linear fits shown for visualization. The plotted subgroup-specific lines are shown for visualization; the cohort-wide correlation reported in the text was weak and non-significant.

Across the cohort, serum 25(OH)D was inversely correlated with CTX (r = −0.14, *p* = 0.141; Figure 3). In stratified descriptive plots, the inverse trend appeared more pronounced in the low-BMD group, whereas the association was essentially flat in the normal-BMD group; however, neither subgroup-specific correlation was statistically significant.

Exploratory mediation analyses evaluating CTX and urinary 8-OHdG/creatinine as potential statistical mediators of the association between serum 25(OH)D and mean CAL yielded very small indirect effects, and the bootstrap confidence intervals crossed zero in both models (Table 6). Specifically, the indirect effect through CTX was −0.0015 (95% CI −0.0054 to 0.0028), and the indirect effect through urinary 8-OHdG/Cr was −0.0016 (95% CI −0.0065 to 0.0021).

In both models, the direct effect of 25(OH)D on mean CAL remained essentially unchanged (−0.0228 and −0.0227, respectively), compared with the total effect (−0.0243), indicating that CTX and 8-OHdG/Cr explained little of the observed inverse association. These mediation analyses should therefore be considered hypothesis-generating.

## 4. Discussion

Postmenopausal women are a demographic characterized by marked alterations in systemic bone metabolism, immune regulation, and inflammatory homeostasis. Estrogen receptors are expressed in periodontal tissues, including gingival fibroblasts, periodontal ligament cells, and alveolar osteoblasts, and estrogen signaling has been implicated in regulating cytokine expression, osteoclastogenesis, and connective tissue turnover [33,34,35,36]. The decline in circulating estrogen at menopause may therefore predispose to a pro-inflammatory, bone-resorptive milieu, heightening susceptibility to periodontal breakdown [37,38,39,40,41].

Our findings reinforce and extend existing evidence that estrogen deficiency, which characterizes the postmenopausal transition, is a substantial modulator of periodontal disease progression and severity.

This cross-sectional study on postmenopausal women with established periodontitis found that low BMD consistently correlates with a more destructive periodontal phenotype. Several objective nutritional biomarkers also tracked the extent of tissue loss. Clinically, women with low BMD exhibited higher mean CAL (2.06 vs. 1.45 mm) and a greater proportion of Stage III disease (34.4% vs. 8.9%), despite similar plaque and BOP levels across BMD groups. This suggests that the observed gradient cannot be easily explained by differences in current oral hygiene practices. These findings support current evidence that osteoporosis/low BMD and periodontitis frequently co-occur after menopause, likely due to shared susceptibility rather than a direct cause-and-effect relationship. Two meta-analyses focusing on postmenopausal women found that osteoporosis or low BMD correlates with worse periodontal health and a higher risk of periodontitis. These studies also highlighted significant heterogeneity across periodontal case definitions, skeletal sites, and adjustment methods [4,6]. A recent narrative review similarly describes the osteoporosis–periodontitis link as clinically significant in older women, emphasizing potentially shared pathways of tissue remodeling and age-related host susceptibility rather than a straightforward cause-and-effect relationship [5]. Our findings extend this by demonstrating that, in a postmenopausal group using 2017 staging, low BMD is associated with both higher average CAL and a significant shift towards Stage III disease, even when plaque and BOP levels are comparable across BMD groups. This supports the concept of shared susceptibility.

Inflammatory regulation in postmenopausal women is further complicated by alterations in innate and adaptive immune responses [42,43]. Estrogen exerts immunomodulatory effects on neutrophil function, macrophage phenotype switching, and T-cell differentiation; its deficiency has been associated with enhanced expression of pro-inflammatory cytokines such as TNF-α, IL-1β, and IL-6 [44,45,46,47,48,49]. Recent transcriptomic analyses in peri-implantitis and periodontitis tissues have identified overlapping inflammatory gene signatures driven by NF-κB and JAK/STAT pathways in estrogen-deficient states [50,51].

The present findings also underscore the variable impact of hormone replacement therapy on periodontal outcomes. While hormone replacement therapy has been shown to attenuate systemic bone loss and reduce circulating pro-inflammatory cytokines, its effects on periodontal parameters have been inconsistent across clinical trials [52,53]. Recent meta-analyses indicate that hormone replacement therapy may confer a protective effect on periodontal tissues, but this effect appears to be moderated by duration of therapy, the timing of initiation relative to menopause onset, and individual risk factors, including smoking and diabetes [54,55]. These nuances highlight the need for more targeted interventional research designed to parse hormonal from behavioral influences on periodontal health in this population.

A noteworthy aspect of our results is the potential role of gene-environment interactions in modulating periodontal susceptibility among postmenopausal women. Polymorphisms in cytokine genes (e.g., IL-1β, TNF-α) and genes involved in estrogen receptor signaling have been associated with differential periodontal responses in estrogen-deficient animal models and human epidemiologic studies [56,57].

This study’s key contribution is the creation of a biomarker-based nutrition phenotype. In models adjusted for robust standard errors and multiple comparisons within the predefined set of nutrition biomarkers, higher serum levels of 25(OH)D, plasma vitamin C, and RBC omega-3 index were each independently associated with lower average CAL. Conversely, ferritin showed no such link, and the BMD × 25(OH)D interaction was not supported. These associations are clinically meaningful: for example, 25(OH)D was inversely related to mean CAL (approximately −0.024 mm per 1 ng/mL), and the omega-3 index showed a similar inverse relationship (around −0.107 mm per 1%), even after adjusting for age, adiposity, glycemia, plaque burden, dental care attendance, and sociodemographic factors. This biomarker-focused approach addresses a common critique in nutrition–periodontology: that diet questionnaires may not accurately capture bioavailability, long-term nutritional status, or individual metabolic differences [58,59,60,61,62].

To aid clinical understanding, emphasizing the magnitude of observed effects is crucial. The adjusted mean CAL is 0.664 mm higher in the low-BMD group, indicating a significant increase in full-mouth periodontal tissue loss. This difference accounts for about 46% of the mean CAL in the normal-BMD group (1.45 mm). While mean CAL provides an overall summary and is not directly equivalent to site-specific CAL thresholds used for staging, such shifts are clinically significant because they indicate a widespread increase in attachment loss throughout the dentition. This may also lead to more sites reaching advanced disease thresholds. Additionally, the biomarker coefficients are clinically relevant: a 15 ng/mL difference in serum 25(OH)D (e.g., 15 vs. 30 ng/mL) is associated with roughly a 0.36 mm decrease in mean CAL. Similarly, a 2% increase in RBC omega-3 index (e.g., 4% vs. 6%) correlates with an approximately 0.21 mm decrease in mean CAL.

The vitamin D findings are consistent with a meta-analysis showing lower serum vitamin D levels in people with periodontitis compared to controls [10]. It also indicates that combining vitamin D supplements with nonsurgical therapy may provide modest improvements in periodontal clinical outcomes. Recent observational studies continue to report that lower serum vitamin D levels track with greater periodontal disease severity, supporting the plausibility of vitamin D as a severity-related biomarker in clinical populations [63,64,65]. Although supplementation trials and observational biomarker studies are not equivalent, their agreement supports biological plausibility, especially in postmenopausal women, where skeletal and alveolar tissue remodeling may be more susceptible to vitamin D deficiency [66,67,68,69].

Similarly, the inverse relationship between plasma vitamin C and mean CAL aligns with a recent systematic review and meta-analysis suggesting that vitamin C supplementation can benefit periodontal health, although results vary by baseline status and other factors [17]. Omega-3 fatty acids are frequently discussed as host-modulatory adjuncts [70,71,72,73], and our biomarker findings are consistent with reviews indicating that adding omega-3s to nonsurgical periodontal therapy can improve CAL and PD outcomes. These effects are influenced by dose, co-interventions, and follow-up [20]. Our findings are also consistent with the broader synthesis by Berg et al. [18], who highlighted converging evidence for the periodontal relevance of micronutrients and omega-3 fatty acids, particularly through mechanisms related to oxidative stress, immune regulation, and tissue regeneration, while noting that biomarker-based and longitudinal studies remain limited.

Conversely, ferritin levels did not correlate with periodontal destruction after adjustments—an important negative finding—implying that ferritin may not be a key factor in periodontal severity. However, caution is warranted since ferritin is biologically pleiotropic and may reflect iron stores or other physiological states [74,75]. Genetic and epidemiologic evidence suggests complex relationships between iron biomarkers, including ferritin, and periodontitis outcomes, indicating that any association between iron and periodontitis is unlikely to be consistent across different indices or populations [76].

From a mechanistic perspective, the results on bone turnover and oxidative DNA damage are consistent but also highlight the limitations of cross-sectional analysis. Although low BMD was associated with higher CTX and higher urinary 8-OHdG/creatinine, the exploratory mediation models yielded only very small indirect effects, with bootstrap confidence intervals crossing zero, and the direct association between 25(OH)D and mean CAL remained essentially unchanged after inclusion of CTX or 8-OHdG/creatinine. Accordingly, these analyses do not support a measurable mediating role for CTX or oxidative DNA damage in this cross-sectional dataset and should be viewed as hypothesis-generating rather than confirmatory.

Regarding oxidative DNA damage, urine biomarkers in periodontitis remain underdeveloped. However, a systematic review confirmed the potential of urine-based markers, highlighting 8-OHdG as a candidate analyte for which research is limited [22]. Although most research on 8-OHdG is context-specific, recent reviews support its role as a marker of oxidative DNA damage relevant to periodontal disease, among other important proinflammatory biomarkers [77,78,79,80,81,82]. In this setting, higher urinary 8-OHdG/creatinine levels in women with low BMD reflect a phenotype associated with remodeling and oxidative stress. Nonetheless, small and non-significant effects observed in exploratory mediation analyses suggest that oxidative stress and bone turnover may partly contribute to the oral–skeletal link but are unlikely to fully explain it in a cross-sectional context.

Several additional limitations need to be highlighted. Firstly, the cross-sectional design prevents us from determining causality: low BMD might lead to more severe periodontal breakdown, but functional changes related to periodontitis (such as mastication and food choices) could also affect nutritional status and, indirectly, skeletal health.

Secondly, this study employs strict exclusion criteria, such as no current or past smoking, no osteoporosis treatment, no menopausal hormone therapy, no recent nutritional supplements, and no major systemic comorbidities. This enhances internal validity by minimizing key confounders that often influence both bone and periodontal health and can significantly impact circulating biomarker profiles. However, it also results in a cohort that is more selective than typical postmenopausal populations, where smoking, anti-resorptive treatments, hormone therapies, and supplement use (like vitamin D, calcium, and multivitamins) are common. Therefore, these findings are most relevant to untreated, relatively healthy postmenopausal women with periodontitis. Further studies across more diverse groups, including treated patients and supplement users, are necessary to confirm the broader applicability of the observed biomarker–periodontitis relationships.

Third, although the sample size was adequate for the primary continuous outcome (mean CAL), the study was not specifically powered to detect small interaction effects; therefore, the non-significant BMD × 25(OH)D interaction mainly argues against large effect modification, while smaller interactions cannot be excluded.

The logistic regression results for severe periodontitis should also be interpreted with caution. Although the observed odds ratio for low BMD was large and directionally consistent with the continuous CAL findings, the number of severe periodontitis events was limited, which can lead to imprecise effect estimates and wider confidence intervals in multivariable logistic models. For this reason, the continuous periodontal outcome (mean CAL) remains the most statistically robust endpoint in the present study.

Lastly, although using objective biomarkers reduces measurement error compared to questionnaires, assessments at a single time point may not reflect long-term nutritional exposures, and residual confounding factors—such as sun exposure, unmeasured dietary habits, lifetime estrogen levels, and access to healthcare—may still influence results. Moreover, the cross-sectional design cannot establish whether nutritional status influences periodontal destruction, whether severe periodontitis affects nutritional status, or both. For example, advanced periodontal disease might impair chewing comfort, alter food texture preferences, and reduce overall dietary quality, which could subsequently alter biomarker levels such as plasma vitamin C and the RBC omega-3 index. This highlights the importance of conducting longitudinal studies with repeated periodontal and biomarker measurements, along with interventional studies focused on nutritional optimization, to better understand the direction of the relationships between nutritional status, BMD, and periodontal tissue loss. These studies are particularly crucial for clarifying reverse causality, such as whether declining periodontal health influences dietary choices and subsequent biomarker profiles.

Future research should focus on longitudinal follow-up to establish the temporal relationship and assess whether baseline nutritional biomarkers predict periodontal progression—such as attachment loss, incident Stage III transitions, or tooth loss—differently across BMD levels. Moreover, since these patients have specific therapeutic needs, a more holistic approach is warranted, both locally and systemically. Since these patients have several comorbidities for which they are medicated, this may influence periodontal disease progression even more [83,84,85,86,87,88,89].

Conducting clinical trials on vitamin D supplementation and omega-3 optimization is warranted to better understand how nutrition affects long-term disease progression. Mechanistic insights could be gained by incorporating assessments of mandibular bone microarchitecture and microbiome profiling to determine whether the association between low BMD and severe periodontitis reflects shared susceptibility to remodeling, changes in microbial ecology, or both. Concurrently, integrating genetic approaches for nutrient-related exposures (e.g., iron indices) can help distinguish causation from confounding in specific biomarker domains.

## 5. Conclusions

In this group of postmenopausal women, lower bone mineral density was linked to more severe periodontal tissue destruction and a greater prevalence of advanced periodontitis. Key nutritional biomarkers—particularly serum 25(OH)D and RBC omega-3 levels—showed independent and inverse relationships with average clinical attachment loss, even after accounting for major confounders and multiple comparisons within the nutrition panel. The observed associations of 25(OH)D with CTX, together with exploratory findings for urinary 8-OHdG, support a biologically plausible oral–skeletal framework linking nutritional status, remodeling activity, and periodontal destruction; however, the cross-sectional design does not allow inference about directionality or causation. These findings underscore the need for longitudinal and intervention research to determine if improving nutritional health can slow periodontal deterioration in women with low BMD.

## Figures and Tables

**Table 1:** Distribution of periodontitis stages (2017 classification) by BMD status.

Periodontitis Stage	Overall (N = 120)	Normal BMD (n = 60)	Low BMD (n = 60)
Stage I	77 (64.2%)	45 (80.4%)	32 (50.0%)
Stage II	16 (13.3%)	6 (10.7%)	10 (15.6%)
Stage III	27 (22.5%)	5 (8.9%)	22 (34.4%)
Stage IV	0 (0.0%)	0 (0.0%)	0 (0.0%)

**Table 2:** Baseline characteristics by BMD status.

Characteristic	Overall (N = 120)	Normal BMD (n = 60)	Low BMD (n = 60)	*p*-Value
Age (years)	62.24 ± 5.50	62.55 ± 5.00	61.97 ± 5.93	0.559
Years since menopause	12.48 ± 6.00	12.98 ± 5.23	12.05 ± 6.60	0.389
BMI (kg/m^2^)	26.79 ± 3.64	25.76 ± 3.56	27.69 ± 3.48	0.004
HbA1c (%)	5.36 ± 0.19	5.34 ± 0.19	5.37 ± 0.20	0.536
Physical activity (IPAQ, MET-min/week)	1345 [973, 2022]	1474 [973, 2056]	1320 [971, 1937]	0.726
Residence (urban)	75 (62.5%)	36 (64.3%)	39 (60.9%)	0.850
Education (university)	39 (32.5%)	22 (39.3%)	17 (26.6%)	0.197
Dental preventive visit ≥ 1/year	70 (58.3%)	35 (62.5%)	35 (54.7%)	0.496
Alcohol use (any)	62 (51.7%)	29 (51.8%)	33 (51.6%)	1.000

Low BMD subgroup composition: osteopenia, n (%) = 56 (87.5); osteoporosis, n (%) = 8 (12.5).

**Table 3:** Periodontal measures, DXA metrics, and biomarkers by BMD status.

Measure	Overall	Normal BMD	Low BMD	*p*-Value
Spine T-score	−0.96 ± 0.88	−0.19 ± 0.40	−1.64 ± 0.58	<0.001
Hip T-score	−0.81 ± 0.85	−0.10 ± 0.43	−1.44 ± 0.60	<0.001
25(OH)D (ng/mL)	20.25 ± 7.26	22.66 ± 7.27	18.14 ± 6.61	<0.001
Plasma vitamin C (µmol/L)	46.35 ± 12.47	49.85 ± 10.43	43.28 ± 13.35	0.003
RBC omega-3 index (%)	4.86 ± 1.20	5.32 ± 1.09	4.45 ± 1.14	<0.001
Ferritin (ng/mL)	61.4 [42.0, 87.9]	61.2 [42.0, 87.1]	61.5 [41.6, 88.3]	0.725
CTX (ng/mL)	0.45 ± 0.14	0.42 ± 0.14	0.48 ± 0.13	0.022
P1NP (ng/mL)	53.95 ± 16.94	51.27 ± 17.18	56.30 ± 16.51	0.106
Urinary 8-OHdG/Cr (ng/mg)	7.5 [5.7, 9.4]	6.8 [5.7, 8.4]	8.2 [5.9, 9.7]	0.030
Teeth present (n)	22.71 ± 3.86	22.73 ± 3.89	22.69 ± 3.86	0.950
Plaque (% sites)	39.76 ± 15.87	40.79 ± 15.49	38.86 ± 16.26	0.506
BOP (% sites)	34.20 ± 10.25	34.06 ± 11.09	34.32 ± 9.55	0.890
Mean CAL (mm)	1.77 ± 0.70	1.45 ± 0.51	2.06 ± 0.72	<0.001
% sites with CAL ≥ 5 mm	1.6 [0.0, 10.2]	0.0 [0.0, 2.4]	6.6 [0.0, 12.3]	<0.001
% sites with PPD ≥ 6 mm	0.0 [0.0, 3.9]	0.0 [0.0, 0.0]	3.0 [0.0, 5.5]	<0.001
Severe periodontitis (Stage III/IV)	27 (22.5%)	5 (8.9%)	22 (34.4%)	0.002

**Table 4:** Adjusted associations with mean CAL (linear regression; robust SE; FDR within nutrition biomarkers).

Predictor	Outcome	β	95% CI	*p*-Value	FDR *q*-Value	Std. β
Low BMD (vs normal)	Mean CAL (mm)	0.664	0.465 to 0.863	<0.001	—	0.477
25(OH)D (ng/mL)	Mean CAL (mm)	−0.024	−0.041 to −0.008	0.005	0.009	−0.253
Plasma vitamin C (µmol/L)	Mean CAL (mm)	−0.016	−0.025 to −0.008	<0.001	<0.001	−0.294
Omega-3 index (%)	Mean CAL (mm)	−0.107	−0.185 to −0.028	0.008	0.011	−0.183
Ferritin (log ng/mL)	Mean CAL (mm)	0.016	−0.162 to 0.194	0.860	0.860	0.013

For clinical interpretation, a 15 ng/mL difference in 25(OH)D corresponds to an estimated 0.36 mm difference in mean CAL, and a 2% difference in RBC omega-3 index corresponds to an estimated 0.21 mm difference in mean CAL.

**Table 5:** Multivariable model for severe periodontitis (Stage III/IV) (logistic regression; robust SE).

Predictor	OR	95% CI	*p*-Value
Low BMD (vs normal)	7.70	2.29–25.90	<0.001
Age (per year)	1.12	1.01–1.24	0.032
BMI (per kg/m^2^)	1.05	0.89–1.24	0.555
Plaque (per 10% sites)	2.00	1.32–3.04	0.001
Preventive dental visit (yes)	0.29	0.08–1.07	0.063
25(OH)D (per 10 ng/mL)	0.16	0.05–0.54	0.003

**Table 6:** Exploratory indirect effects of 25(OH)D on mean CAL via mechanistic markers.

Mediator	Indirect Effect a × b (95% CI)	Direct Effect	Total Effect
CTX (ng/mL)	−0.0015 (−0.0054–0.0028)	−0.0228	−0.0243
Urinary 8-OHdG/Cr (ng/mg)	−0.0016 (−0.0065–0.0021)	−0.0227	−0.0243

## Data Availability

The original contributions presented in this study are included in the article. Further inquiries can be directed to the corresponding author.

## Abbreviations

The following abbreviations are used in this manuscript:

25(OH)D	25-hydroxyvitamin D
8-OHdG	8-hydroxy-2′-deoxyguanosine
BMD	Bone mineral density
BMI	Body mass index
BOP	Bleeding on probing
CAL	Clinical attachment level
CEJ	Cemento–enamel junction
CI	Confidence interval
CTX (β-CTX)	C-terminal telopeptide of type I collagen
DHA	Docosahexaenoic acid
DXA	Dual-energy X-ray absorptiometry
ELISA	Enzyme-linked immunosorbent assay
EPA	Eicosapentaenoic acid
FDR	False discovery rate (Benjamini–Hochberg procedure)
GC	Gas chromatography
HbA1c	Glycated hemoglobin
HC3	Heteroskedasticity-consistent (HC3) robust standard errors
HPLC	High-performance liquid chromatography
ICC	Intraclass correlation coefficient
IPAQ-SF	International Physical Activity Questionnaire—Short Form
IQR	Interquartile range
ISCD	International Society for Clinical Densitometry
MET	Metabolic equivalent of task (reported as MET-min/week)
OR	Odds ratio
P1NP	Procollagen type I N-terminal propeptide
PPD	Probing pocket depth
RBC	Red blood cell
RBL	Radiographic bone loss
SD	Standard deviation
SERMs	Selective estrogen receptor modulators
SNRIs	Serotonin–norepinephrine reuptake inhibitors
SSRIs	Selective serotonin reuptake inhibitors
STROBE	Strengthening the Reporting of Observational Studies in Epidemiology
UNC-15	University of North Carolina 15 mm periodontal probe (“UNC-15” probe)
UV	Ultraviolet
